# Predictive Processing and the Representation Wars

**DOI:** 10.1007/s11023-017-9441-6

**Published:** 2017-06-26

**Authors:** Daniel Williams

**Affiliations:** Faculty of Philosophy, Trinity Hall, University of Cambridge, Cambridge, UK

**Keywords:** Predictive processing, Clark, Mental representation, Representation wars, Intentionality, The job description challenge, The free-energy principle, Organism-relativity, Structural resemblance

## Abstract

Clark has recently suggested that predictive processing advances a theory of neural function with the resources to put an ecumenical end to the “representation wars” of recent cognitive science. In this paper I defend and develop this suggestion. First, I broaden the representation wars to include three foundational challenges to representational cognitive science. Second, I articulate three features of predictive processing’s account of internal representation that distinguish it from more orthodox representationalist frameworks. Specifically, I argue that it posits a resemblance-based representational architecture with organism-relative contents that functions in the service of pragmatic success, not veridical representation. Finally, I argue that internal representation so understood is either impervious to the three anti-representationalist challenges I outline or can actively embrace them.

## Introduction

Predictive processing is an ambitious theory in cognitive and computational neuroscience. Its central thesis is that brains self-organize around the imperative to minimize a certain kind of *error*: the mismatch between internally generated, model-based predictions of their sensory inputs and the externally generated sensory inputs themselves (Clark 2016; Friston 2009, 2010; Hohwy 2013). Clark (2015) has recently suggested that this overarching theory of neural function has the resources to put an ecumenical end to what he calls the “representation wars” of recent cognitive science. Specifically, he argues that it implies an understanding of internal representation that can accommodate important insights from the *enactivist* tradition without renouncing the theory’s representational credentials.

In this paper I defend and develop Clark’s suggestion. First, I broaden the representation wars beyond those that have characterised the enactivist debate. I outline three important challenges to representational cognitive science advanced by a motley crew of pragmatists, behaviourists, reductionists, and those in the tradition of embodied, embedded, extended and enactive cognition. Second, I articulate three features of predictive processing’s account of internal representation that distinguish it from more orthodox representationalist frameworks. Specifically, I argue that it posits a *resemblance*-*based* representational architecture with *organism*-*relative* contents that functions in the service of *pragmatic success*, not veridical representation. Finally, I argue that internal representation *so understood* is either impervious to these three anti-representationalist challenges or can actively embrace them.

The structure of the paper is as follows. In Sect. 2 I identify three foundational challenges to representational cognitive science, concerning (1) representational *function*, (2) representational *content*, and (3) *cognitive* function. In Sect. 3 I provide a brief introduction to predictive processing and elaborate its account of internal representation. In Sect. 4 I argue that this account of internal representation can either *accommodate* or *avoid* the concerns enumerated in Sect. 2.

## The Representation Wars

The concept of internal representation is central to the contemporary cognitive sciences and has been since the downfall of behaviourism and the “cognitive revolution.” A foundational assumption across these sciences is that intelligent behaviour and adaptive response mandates the construction and manipulation of content-bearing internal states or *stand*-*ins* for elements of the distal environment (Bermudez 2010; Von Eckardt 2012).

Despite this orthodoxy, the attribution of representational states has always been mired in controversy and confusion. As Dietrich (2007, 1) puts it, “though there is a vast quantity of on-going research dependent on representations… no scientist knows how mental representations represent,” a state of affairs that “has persisted since the inception of the cognitive sciences.” Stubborn worries concerning the *metaphysics* of representation, the nature of representational *explanation* and the apparent theoretical limits of traditional cognitive science have provoked outright scepticism towards internal representations in various heterodox corners of psychology and philosophy.

Since at least the early 1990s, a significant source of this scepticism has been the tradition of embodied, embedded, enactive and extended (henceforth EEEE) cognition. Members of this movement have argued that the concept of internal representation should be marginalised or even eliminated in the sciences of mind and behaviour (cf. Anderson 2014; Chemero 2009; Hutto and Myin 2013; Varela et al. 1993).^1^

The resultant debates have some claim to be called the “representation wars,” both for the sharp divisions they’ve sown concerning the status and proper extent of representational explanation, and for their apparent resistance to straightforward, empirical resolution. To frame them as a recent phenomenon, however, is to ignore the extent to which many of the core bones of contention go back much further in the history of psychology and philosophy, finding their first expression in the work of pragmatists, behaviourists, and physicalist reductionists not necessarily supportive of the positive research agendas in the EEEE tradition.

In this section I give a brief overview of three of these historic and foundational challenges to representational cognitive science. The aim is not to be exhaustive, to adequately defend these sceptical challenges or to consider the myriad responses to them advanced in the literature over many years—an impossible ambition in a paper of this scope. Rather the hope is to identify three *very general* sources of scepticism concerning the existence and extent of internal representation in cognition, and reveal the way in which superficially different kinds of anti-representationalism have been motivated by an underlying stock of core grievances. These foundational concerns, I think, have a good claim to have laid the framework for what might reasonably be called the representation wars. They concern representational *function*, representational *content*, and *cognitive* function.

### Representational Function

The first challenge asserts that the concept of representation implies a functional role that the physical structures and processes implicated in intelligence either *do not* or *cannot* perform. Variants on this challenge thus hinge on two variables: first, a specification of what the relevant functional role is—that is, what characteristics an internal structure must possess to qualify as genuinely representational;^2^ and second, a specification of what the relevant physical structures and processes implicated in intelligence are. Two prominent examples of this anti-representationalist strategy are worth briefly reviewing.

First, many hold that an internal representation’s *content* (or the properties in virtue of which it possesses that content) must be causative for it to qualify as genuinely representational (O’Brien and Opie 2004; Ramsey 2007). As Dretske (1988, 80) puts it, “the fact that [representations] have a content, the fact that they have a semantic character, must be relevant to the kind of effects they produce.” A plausible motivation for this functional claim is this: if internal representations are to genuinely *explain* intelligent behaviour, their *effects* on behaviour must be a function of their representational *status* as content-bearers. Without this, a *representational* explanation of the system’s behaviour would be causally redundant. Anti-representationalists then seize on this functional consideration to argue for an ontological conclusion. Stich (1983), for example, famously argues that the causal irrelevance of content to classical computational architectures implies at best a “syntactic” theory of mind, not a representational one.^3^

Second, many theorists have argued that representation implies a *triadic* relation between the *vehicle*, its *target*, and—crucially—the cognitive system that *uses* or *interprets* the former to direct its behaviour appropriately towards the latter (O’Brien 2015; Ramsey 2007; Von Eckardt 2012). One rationale for this functional claim is that it is implied by the very concept of representation.^4^ A deeper rationale, however, resembles the requirement that content be causative: for an internal structure to qualify as representational—for it to perform interesting work *qua* representation—its representational status must surely be *exploited* by the cognitive system of which it is a part (O’Brien 2015; Shea 2014). In the mid-twentieth century, this functional claim was taken to imply that cognitive processes could not implicate internal representations without inner homunculi as intelligent as the processes they were drawn upon to explain (Ryle 1949; Wittgenstein and Anscombe 1953).^5^

What should we make of arguments like this? As noted above, they come in stronger and weaker forms. The strongest conclusion is that the concept of representation is so mired in folk superstition and ways of thinking that it deserves no place in mature science (Rosenberg 2011, 2015). This claim typically relies on a very strong form of physicalist reductionism, however, or else threatens to define the concept of internal representation out of existence.

A weaker and more plausible manifestation comes in the form of a *challenge*—specifically, what Ramsey (2007) calls the “job description challenge.” Drawing on the above considerations, the challenge is to demonstrate that the relevant component parts and operations of cognitive systems perform recognisably representational *jobs*—that their status *as representations* genuinely explains the cognitive system’s behaviour.

Anti-representationalists who pursue this line of argument contend that the challenge is not met. Ramsey (2007) himself, for example, argues that the concept of representation has been trivialized in contemporary cognitive science, and that many of the structures *characterised* as representations in our most promising approaches to cognition are not usefully understood as performing representational roles. A complementary strategy is developed by those who seek to model the physical structures and processes responsible for intelligence within a non-representational framework—an attempted existence proof that there is nothing distinctively representational about the functions they perform (Anderson 2014; Chemero 2009).

It is not my intention to evaluate these arguments here. Before moving on, however, I note something that will be a recurring theme in each of the three challenges I outline: except for the very strong manifestation of this challenge, its application is not *global*. That is, one might agree with Ramsey that the concept of representation in contemporary cognitive science has been trivialised, and that many of the processes characterised as representations are not genuinely representational, while nevertheless thinking that *some* theories do genuinely posit full-blooded representational structures.^6^

### Representational Content

The second challenge to representational cognitive science is the most notorious. It contends that representational *content* cannot be placed within a naturalistic metaphysics, and so does not exist.

This scepticism has given rise to what Von Eckardt (2012) calls “the foundational problem of cognitive science,” the “content determination problem”: is it possible to identify the natural properties, relations and processes that determine the intentional properties of internal representations without circularity? A widely-held assumption is that unless this problem can be answered—unless content can be reduced to naturalistically kosher non-content—representational *explanation* in cognitive science must at best be an instrumental gloss on fundamentally non-representational processes (Fodor 1987; Sterelny 1991). Quine (1960) most famously advanced this scepticism, but it has surfaced repeatedly in different guises over the past half a century or so (cf. Hutto and Myin 2013; Rosenberg 2015). Importantly, the challenge is *posterior* to the foregoing worry about representational *function*: scepticism about *content* can only arise for states or structures assumed to be functioning as content-bearing representations in the first place (Ramsey 2007).

Much of the work attempting to answer this challenge has taken place within the framework of “naturalistic psychosemantics,” where the goal is to account for the reference and extension of in-the-head symbols from which the propositional contents of intentional states can be recursively constructed (Fodor 1987).^7^ This project is motivated by at least three considerations. First, a popular view is that cognition is rule-governed symbol manipulation and that much of human and complex nonhuman animal cognition takes place within a discrete symbol system with a combinatorial syntax and semantics—that is, a *language* (Fodor 1975; Schneider 2011). Second, folk psychology and its postulation of propositional attitudes licenses the characterisation of mental states with the semantic vocabulary we bring to bear on language (Sellars et al. [1956] 1997). Finally, truth-conditional semantics suggests a systematic theory of content for such a symbol system, whereby truth-conditions of molecular symbol structures (the contents of propositional attitudes) are constructed from the reference and extension of their constituents and their mode of combination (cf. Davidson 1967).

The challenge is thus to account for the reference mapping from in-the-head symbols to things in the environment, subject to the strictures of some form of metaphysical naturalism and a host of further theoretical desiderata—determinacy, shared contents, and the possibility of misrepresentation, for example—that have proven stubbornly difficult to satisfy. Anti-representationalists who pursue this line of argument contend that the challenge cannot be met: meaning is perhaps an ineliminable part of our folk ontology—an “adaptive fiction,” as Rosenberg (2015) puts it—but deserves no place in literal science.

Again, it is not my intention to evaluate this form of scepticism here. As before, however, I flag up a general lesson: insofar as the bulk of interest in this area has fallen on *linguaformal* semantic properties and the preservation of folk psychological intuitions, it invites the possibility that one might *embrace* this form of scepticism while nevertheless thinking that there are robust kinds of internal representation *not* properly characterised with the semantic vocabulary appropriate to language or hostage to folk intuition, and thus not vulnerable to the same kinds of challenges (c.f. Churchland 2012; Cummins 1996; O’Brien and Opie 2004, 2015). I return to this important point in Sect. 4.

### Cognitive Function

Whatever one thinks of the foregoing challenges, they are relatively well-defined. The same is not true for the third I will consider. Nevertheless, it has played an important historical role in the anti-representationalist tradition and continues to exert a considerable influence in discussions concerning the existence and extent of internal representations today. The challenge has two parts. The first is summarised in the slogan that “cognition exists to guide action” (Glenberg et al. 2013, 573)—or, in, Anderson’s (2003, 92) words, that “thinking beings ought…[to] be considered first and foremost as acting beings.” The second is an implication often drawn from this slogan—indeed, often not *distinguished* from it in the embodied cognition literature—that the concept of internal representation should therefore be either marginalised (Anderson 2014) or eliminated (Chemero 2009) in the cognitive sciences. I consider both stages in turn.

First, then, the idea that we should understand the nature of thought in terms of its role in guiding action of course goes back to the American pragmatists, as does the broadly anti-representationalist conclusion drawn from it (cf. Godfrey-Smith 2015). It is a package of commitments nicely encapsulated in the Deweyan slogan popularised by Rorty (1979, 1989) that mind and language are for “coping, not copying.”

It is only in recent decades, however, that this action-oriented perspective has become a defining theme of a scientific research programme. The tradition of ecological psychology initiated by Gibson (1979), for example, “takes as its *starting assumption* that animals’ perceptual systems are geared for *action in the world*—foraging, finding shelter, avoiding predators and the like—and not to creating a replica of the world inside their heads” (Barrett 2011, 155, my emphasis). More generally, the thesis that “cognition is for action” is often advanced by scientists and philosophers working within the EEEE tradition as a subversive claim with destructive implications for traditional representationalist conceptions of the mind. Engel et al. (2015, 1), for example, argue that cognitive science is experiencing a “paradigm shift” in the form of a “*pragmatic turn* away from the traditional representation-centred framework” towards a view that understands cognition “as subserving action.”

The slogan that “cognition is for action,” however, can seem hopelessly vague (Goldinger et al. 2016). It is often justified on broadly evolutionary grounds, as with Anderson and Chemero’s (2016) claim that “the brain evolved *to* guide action”. But—*prima facie,* at least—an evolutionary perspective would suggest that the brain evolved to facilitate *survival and reproduction*, a purpose plausibly served by an amalgam of different functions for different organisms under different environmental conditions, and thus unlikely to be illuminated by a conception of “action” or “action-relatedness” so broad to encompass them all.

A popular response to this worry is to explicate “action-oriented views” in terms of *regulation* and *control* (Anderson 2014; Cisek 1999; Van Gelder 1995). This control-theoretic perspective takes on various forms among different authors in the EEEE tradition, but the core idea is that a brain should first and foremost be understood as a “control system for the [organism’s] interaction with the external environment” (Pezzulo 2016, 24). Cisek (1999) has developed this view in interesting and influential ways, drawing on insights from Dewey, mid-twentieth century cybernetics and perceptual control theory. He notes that living systems are distinguished from non-living systems in acting upon their environments to regulate their essential variables and thus maintain internally optimal states. In this way they effectively self-organize and thus “actively, if temporarily, resist entropic dissolution” (Anderson 2014, 183). He then takes this fact to imply a fundamental job description for brains: “to exert control over the organism’s state within its environment” (Cisek 1999, 8–9) and thus “maintain organism-relevant variables within some desired range” (Anderson 2016, 7). Viewed in this light, one sees that “the fundamental cognitive problem facing the organism—deciding what to do next—is best understood not as choosing the right response to a given stimulus, but rather as choosing the right stimulus—the right experience to seek—in light of a goal” (Anderson 2016, 7).

This control-theoretic perspective on brain function is not implausible, and is supported by a growing body of work in theoretical biology and neuroscience (Barrett 2017a; Sterling and Laughlin 2015). Indeed, as we will see in Sect. 3.4, it is a central tenet of the predictive processing framework to be defended here. Suppose one accepts it, however. Why should it constitute a threat to representationalist theories of cognition? Which view is it opposed to?

The enemy here is a “reconstructive” understanding of perception and cognition alleged to be characteristic of classical cognitive science, according to which “the purpose of perception is to build objective models (representations) of the mind-independent world” (Anderson 2017, 5). On this “representation-centred paradigm,” cognition is “understood as a capacity for deriving world-models, which might then provide a database for thinking, planning, and problem-solving” (Engel et al. 2015, 3). The “aim of the brain” is thus to internally *reconstruct* the objective structure of the surrounding environment in the form of “observer-independent” (Anderson 2014, 172) or “neutrally specified models” (*ibid* 191) for the purposes of “higher cognition,” such that “the *subject* of cognition is a detached observer with a “bird’s eye” view of the world” (Engel et al. 2015, 2).

According to Anderson and other advocates of embodied cognition, this reconstructive understanding of brain function cannot be sustained once we recognise that it “evolved to control action.” Specifically, an action-oriented perspective on cognition forces us to recognise the many profound ways in which contingent and idiosyncratic contributions of the organism—its practical interests, morphology, response profile, and so on—are implicated in all aspects of cognitive functioning. This influence is not well-described by the concept of *re*-*presentation* and its associated implication that internal states *mirror* or *reflect* independently identifiable contents of the external world—what Varela et al. (1993) call a “pre-given world.” An action-oriented perspective implies “not a representational but a *performative* theory of mind and brain” (Anderson 2014, 162), in which “neuroscience would not need to explain how brains act as world-mirroring devices but rather as ‘vehicles of world-making’ (Varela et al. 1993): vehicles which support, based on individual learning history, *the construction of the experienced world* and the guidance of action” (Engel et al. 2015, 4, my emphasis).

This idea is of course central to the tradition of ecological psychology, where the concept of “affordances” in characterising perception emphasises idiosyncratic properties of the animal and the “abilities available in… [its] form of life” in structuring its responsiveness to environmental conditions (Ramstead et al. 2016, 16). On this view, perception relates an organism to its “Umwelt” (von Uexküll [1934] 1957), a reality fundamentally warped around its practical interests and morphology (Barrett 2011, 80). It is also the dimension of the enactivist tradition that Clark seeks to placate in his treatment of predictive processing. Specifically, enactivists deny that the function of “perception is… to determine how some perceiver-independent world is to be recovered” in the form of “action-neutral” representations (Varela et al. 1993, 173–174).^8^ As Clark (2016, 293) puts it, they advance in opposition to this representationalist view a perspective in which “organism and world… are… co-defined by a history of structural coupling: a kind of active “fitting” of each to the other, rather than a passive “mirroring”.”

How plausible is this line of argument? As a rebuke to an unfortunate tendency in both the philosophical tradition and classical cognitive science of viewing the mind as something that floats free of the organism’s time-pressured practical engagements with the environment, it is salutary. The idea of what Wilson (2002) calls “representation for representation’s sake” *is* biologically unrealistic, and the presumption that cognition consists in the construction and manipulation of what Anderson (2014) calls “neutrally specified” or “observer-independent” models does suggest an implausibly passive conception of brain function—a perspective nicely captured by what Dewey (1925) decried as the “spectator theory of knowledge.”

Nevertheless, to visit our recurring theme once again, it is unclear why these important considerations should be taken to undermine internal representation *as such*. After all, the claim that cognition is for “coping, not copying” is evidently consistent with the view that an enormous amount of the latter occurs in facilitating the former. And the fact that the brain’s internal states do not comprise “objective” or “impartial” models of the distal environment does not entail that they do not comprise internal models at all.

### Summary

The foregoing overview has provided a skeletal, whistle-stop tour of three foundational challenges to representational cognitive science that have emerged in the previous century. There is an enormous amount of work enumerating and answering these challenges that I have ignored here. Further, I have focused predominantly on destructive *challenges* to representationalism, rather than the important body of constructive anti-representationalist research programmes such challenges have given rise to.

One important lesson that I have tried to stress in each of these challenges, however, is this: what can often seem like a challenge to representational cognitive science *as such* emerges on closer inspection to be an objection to one specific kind of internal representation, or to a specific interpretation of what internal representation amounts to. This suggests the possibility of an ecumenical resolution of the theoretical divisions these challenges have sown—one which embraces internal representations but which nevertheless does justice to the foregoing concerns. This hopeful prospect, of course, is the thesis advanced by Clark (2015) that I wish to defend here, and brings me to predictive processing.

## Predictive Processing and Internal Representation

There are numerous excellent introductions to predictive processing of different levels of mathematical sophistication and from different theoretical perspectives in both the scientific and philosophical literature.^9^ The aim of this section is twofold: to give a brief and selective introduction to its central claims and core theoretical structure, and to articulate the account of internal representation that falls out of it. I postpone consideration of how this account answers the foregoing anti-representationalist challenges until Sect. 4.

### Predictive Processing: A Brief Overview

First, then, predictive processing shares with mainstream cognitive science the following assumption:^10^ to generate adaptively valuable behaviour in real time, brains must identify the evolving state of the environment—including the internal, bodily environment—from the trace of ambiguous input signals it leaves on the organism’s sensorium (Clark 2013). These sensory inputs are *ambiguous* in that they dramatically underdetermine their environmental causes. Further, adaptive behaviour mandates the *integration* of potentially conflicting sensory cues from *across* the perceptual modalities as well as some means of coping with the ineliminable noise that arises in biological systems (Rescorla 2013, 2016). In other words, brains confront an almost unimaginably difficult *causal inference problem*: they must infer the hidden state of the constantly changing environment from its profoundly non-linear and ambiguous effects on the organism’s numerous sensory transducers (Hohwy 2013).

A popular approach in perceptual psychology and neuroscience models this process of causal inference as *Bayesian inference*, an optimal way of combining *prior expectations* based on learning or innate endowment with incoming evidence to arrive at an estimate of how things are (Lee and Mumford 2003; Penny 2012; Rescorla 2013). This Bayesian approach has numerous well-advertised attractions: it provides a compelling account of how perceptual systems overcome the noise and ambiguity in their sensory inputs, and offers illuminating explanations of otherwise perplexing phenomena such as perceptual *constancies* and *illusions* (cf. Rescorla 2013). In addition, there is extensive behavioural evidence that subjects do in fact integrate perceptual cues in this Bayes optimal way (cf. Knill and Pouget 2004).

Thus specified, however, the “Bayesian brain hypothesis” (Knill and Pouget 2004) is a purely “performance-oriented model”: it asserts *that* the brain performs Bayesian inference without explaining *how* it does so. What is needed is a specification of the actual brain-based algorithms that realise Bayesian inference and the neural structures and processes that implement them (Colombo and Seriès 2012).

*Predictive processing* attempts to bridge this gap (Friston et al. 2017). It claims that (approximate) Bayesian inference occurs through hierarchical predictive coding and prediction error minimization. There are two concepts central to an understanding of this process: the concept of a *hierarchical probabilistic generative model*, and the concept of *predictive coding*. I introduce both in turn.

A *generative model* represents the hidden, interacting causes (the latent variables) responsible for generating some data set, and can induce candidate instances of that data for itself based on its generative assumptions (Danks 2014, 44; Hinton et al. 1995). This is the data *expected* given its model structure and parameters: its representation of the causal matrix currently responsible for its inputs. In this way a generative model can be contrasted with a purely *discriminative* model that maps input data onto appropriate categorisations of that data, familiar from the first wave of feed-forward connectionist models (cf. Rumelhart and McClelland 1986).

Crucially, generative models for rich, structured bodies of data must be multilevel or hierarchical, separating out hidden causes at different levels of abstraction (Clark 2013; Hinton et al. 1995). A generative model for vision, for example, might represent the causal matrix responsible for the evolving stream of retinal stimulation at different levels of spatial and temporal scale. Levels low down in the hierarchy (e.g. in V1) will thus represent fast-moving regularities or constancies implicating fine-grained environmental features (e.g. light distributions, orientation, shading, and so on) while levels higher up will estimate slower-moving regularities involving more invariant conditions (Friston 2008; Hohwy 2013, 27). Crucially, this means that the data for every level of the hierarchy—*with the exception of the first*—consists of representations at the level below, ensuring that deeper “layers of neural populations produce increasingly abstract statistical summaries of the original visual input” (Blouw et al. 2016, 6).

Finally, a hierarchical generative model is *probabilistic* if these representations throughout the model are realised as probability distributions or density functions—that is, representations of the probability that a random variable (or set of variables) assumes a given value (Knill and Pouget 2004, 712). We saw above that perceptual inference is ineradicably saturated with uncertainty. Hierarchical probabilistic generative models (henceforth HPGMS) factor in this uncertainty, encoding probability distributions defined over distal, interacting causes at multiple levels of abstraction.

*Predictive coding* then characterises the nature of message-passing throughout this hierarchical generative model. Traditional approaches in perceptual psychology and neuroscience model perception as a process of bottom-up *feature detection* or *evidence accumulation* (e.g. Marr 2010). Roughly, perceptual systems detect increasingly sophisticated features of the environment as information passes from initial sensory inputs up through the relevant area of sensory cortex. Predictive coding reverses this picture. Descending predictions carried from top-down synaptic connections are issued from higher levels of cortical hierarchies, reflecting the sensory data the brain expects given the state of its generative model. These predictions are compared against the sensory data or the representation at the level below, and the only information then passed back up the hierarchy is the *mismatch* between the two distributions: a *prediction error* quantifying the divergence between the sensory data the model *expects* (at each level) and the data it *receives* (Lee and Mumford 2003).

By combining generative models and predictive coding in this way, the brain can identify the multilevel set of interacting hidden causes that best *explain* its evolving sensory input by *minimizing the error* in its *predictions* of this input—a process thought to combine *prior* expectations enshrined in the generative model with incoming evidence in the Bayes optimal way outlined above (Clark 2013; Hohwy 2013). In an inversion of traditional wisdom, sensory input is thus harnessed as *feedback* to the brain’s endogenously generated predictions. Crucially, transforming sensory input into *feedback* in this way enables brains to *learn* the generative models that facilitate the effective minimization of prediction error by… *minimizing prediction error*. That is, brains induce both the structure and parameters of the generative model that makes Bayesian inference possible by reconfiguring their patterns of neuronal connection in response to errors in their predictions of the incoming sensory data, such that both learning and online response are governed by the same overarching principle (Clark 2016, 15).

So far this process is extremely reactive. An overarching imperative to minimize prediction error, however, can be satisfied in one of two ways: either by updating top-down predictions to bring them into alignment with the incoming data, or by *updating the incoming data to bring it into alignment with top*-*down predictions*. Whereas the former constitutes “reactive inference” (Sims 2016), the latter is known as “active inference” (Hohwy 2013). The upshot is that “perceiving and acting are but two different ways of doing the same thing” (Hohwy 2013, 76).

One manifestation of active inference is *sensory sampling*: the brain actively moves the sensory organs around to confirm (or disconfirm) its model-based predictions of current environmental state (Hohwy 2013, 75–82). In the most ambitious formulation of predictive processing to be considered here, however, active inference is extended to explain what would ordinarily be thought of as “goal-directed” behaviour. On this view, motor control is a matter of predicting the proprioceptive sensory inputs the brain would receive were the body configured in a desired way. The resultant prediction error conditioned by the absence of this action is then quashed in a self-fulfilling prophecy: the motor plant activates classical reflex arcs to bring the incoming signal into alignment with top-down proprioceptive predictions (Friston et al. 2017). More generally, the “goals” that ultimately drive such behaviour are assumed to be conditioned by *interoceptive* predictions that function as homeostatic set-points—a crucial feature of the framework that I return to in more depth in Sect. 3.4 (Seth 2015; Seth and Friston 2016).

For now, however, this emaciated summary of predictive processing must do. There are numerous dimensions of the theory I have had to leave out in the foregoing presentation, not least the crucial role of “precision-weighting” throughout the predictive processing architecture, in which the influence of sensory and prior information is modulated at every level by estimates of their context-variable reliability (i.e. *precision*) (cf. Clark 2016, ch. 2). Further, there is a large and growing literature extending this basic framework to explain an array of other psychological phenomena: attention (Feldman and Friston 2010), social cognition (Friston and Frith 2015), neural pathologies such as schizophrenia (Fletcher and Frith 2008) and autism (Van de Cruys et al. 2013), language (Lupyan and Clark 2015), off-line forms of cognition such as dreaming, mental time-travel, and counterfactual reasoning (cf. Clark 2016, ch. 3), and more.

Thanks both to this wealth of fertile theoretical applications and its connection to deeper considerations drawn from theoretical biology to be expanded below (in Sect. 3.4), many advocates of predictive processing are confident that it heralds a genuine “paradigm shift in the cognitive neurosciences” (Friston et al. 2017, 1)—“the most complete framework for date for explaining perception, cognition, and action” (Seth 2015, 1). As Hohwy (2017, 1) puts it, the upshot is a “unified theory of brain function [that] seeks to explain all aspects of mind and cognition as the upshots of prediction error minimization” (Hohwy 2017, 1). Next I turn to consider in more depth the account of internal representation that falls out of this unified theory of brain function.

### Predictive Processing and Internal Representation

*Prima facie*, at least, predictive processing is a robustly representational theory of cognition. At its core is the notion of a hierarchical generative *model* estimating the most probable causes of the brain’s evolving sensory inputs. In this section I identify three features of its account of internal representation that distinguish it from more orthodox understandings of internal representation. In the next section I argue that this account can either accommodate or avoid the anti-representationalist challenges enumerated in Sect. 2.

### The Model-Building Brain

First, in stark opposition to much of classical cognitive science and contemporary philosophy, predictive processing’s account of internal representation is resolutely non-*linguaformal*.^11^

A popular view—what Horst (2016) calls the “standard view” in classical cognitive science and philosophy—holds that the bulk of human cognition takes place within a system of representation characterised by the structural units, semantic properties and forms of reasoning associated with language (Fodor and Pylyshyn 2015). On this view, a three-tiered compositional architecture of word-sized concepts, sentence-sized intentional states and argument-sized inferences provides the central medium of brain-bound representation and computation, interfacing with peripheral perceptual input modules and motor output modules (Fodor and Pylyshyn 2015; Schneider 2011). The upshot is that the “fundamental unit of cognition is the *judgement*, a unit that lives in a space of sundry logical relations with other actual and possible judgements, a unit that displays the characteristic feature of truth and falsity” (Churchland 2012, 4).

A long, alternative tradition in philosophy and psychology rejects this propositionalist account of mental representation in favour of an *iconic* or *analogue* understanding of the mind’s representational capacities (O’Brien and Opie 2004, 2010, 2015). Advocates of this view contend that much of sophisticated internal representation is founded on *similarity* or *physical analogy* with the mind’s objects. Instead of language, the relevant paradigms from everyday life here are representational tools such as pictures, diagrams, graphs, maps, and models. Here is Craik (1943), for example, eerily prefiguring central themes of predictive processing in the early 1940s:

“If the organism carries a “small-scale model” of external reality… within its head, it is able to… react to future situations before they arise, utilize the knowledge of past events in dealing with the present and future, and in every way react in a much fuller, safer, and more competent manner to the emergencies which face it” (Craik 1943, 61).

In predictive processing, Craik’s hypothesised “small-scale model” becomes the brain’s rich, hierarchically structured generative model of hidden bodily and environmental causes. This generative model functions as a “physical working model” realised in cortical networks that “shares a *relation*-*structure* to that of the process it imitates” (Craik 1943, 51, my emphasis). Specifically, it recapitulates the *causal*-*probabilistic structure* of dependence relationships among functionally significant (see below) environmental variables as revealed in the statistical patterns of sensory input. In this way “neuroanatomy and neurophysiology can be regarded as a distillation of statistical or causal structure in the environment disclosed by sensory samples” (Seth and Friston 2016, 3), and the brain’s generative model “inherits the dynamics of the environment and can predict its sensory products accurately” (Kiebel 2009, 7). In other words, brains can only generate “from the inside” successful anticipations of the sensory signals produced by the environment by *becoming that environment.*

If this is right, it suggests the brain and its environment would comprise two dynamical systems whose evolutions *in interaction with each other* could be (*roughly*—see below) represented by the same set of differential equations (Wiese 2016, 12). For this reason, Gladziejewski (2015) argues that generative models within predictive processing function as “causal-probabilistic maps”: structural models comprised of states whose functional relations roughly recapitulate the dynamical interactions among their represented objects.^12^ The upshot is that

“the hierarchical structure of the real world literally comes to be “reflected” by the hierarchical architectures trying to minimize prediction error, not just at the level of sensory input but at all levels of the hierarchy” (Friston 2002, 237–238; see also Friston 2005, 825).

This relation of “second-order structural resemblance” (O’Brien and Opie 2004, 2010, 2015) is familiar from graphical models in machine learning and statistics (Pearl 2009).^13^ Indeed, structural resemblance between the representation and its domain is plausibly a characteristic of *all* statistical models—in fact, of all models (Giere 2004; Godfrey-Smith 2006). A Bayesian network, for example, consists of a structure of variables and their causal-probabilistic dependencies, along with a set of model parameters that determine the relevant strengths of such relationships (Pearl 2009, 13–20). If accurate, this causal-probabilistic structure will replicate the causal-probabilistic structure of the domain it represents. Further, as with predictive processing, such networks can be *learned* by comparing the data *they* generate with the data generated by the domain itself (Danks 2014, 44).

Predictive processing in effect bets that the neocortex instantiates a hierarchical Bayesian network (Gladziejewski 2015, 571). However, instead of explicitly *representing* the parameters that determine the strength of the relevant causal-probabilistic dependencies among environmental variables—in a set of symbolic descriptions, for example—cortical networks *instantiate* such dependencies in the configurations of synaptic connections that govern neuronal activity (Hinton 2005). In so doing they effectively realise a dynamical model (albeit a causal-probabilistic one) of the body and environment. An interesting upshot is that—*if* predictive processing is correct—brains deploy the very kind of representation that advocates of dynamical systems theory argue *we* should use to model the brain.^14^

Of course, this is highly schematic, and much more work needs to be carried out here, both in clarifying the relevant relation of resemblance, and in explaining the mechanics of how patterns of neuronal activity can mimic environmental dynamics.^15^ Nevertheless, it emphasises something important about generative models within the context of predictive processing: their description as “models” should be construed quite literally. They are physical structures that structurally resemble their targets. If the hype surrounding predictive processing is well-founded, it suggests that the pendulum in coming years might swing back towards (structural) resemblance accounts of mental representation that have in recent times proven very unpopular in the philosophy of mind (c.f. Cummins 1996; Horst 2016). That is, we might finally have a compelling alternative to language-like accounts of our fundamental cognitive architecture.^16^

The upshot is a beautifully Aristotelian picture of the mind as an organ *enformed* by the dynamical structure of the environment it interacts with. The brain emerges as an arena not for the construction and manipulation of internal judgements but as a generator of “causal-probabilistic maps.”

### The Pragmatic Brain

The overarching function of predictive brains is the minimization of prediction error. In many introductions to predictive processing in the literature, you could be forgiven for thinking this is simply a good trick for learning and updating a model of the world in the service of veridical causal inference and effective intervention. Indeed, it was heuristically useful to introduce the framework in a similar way above. On this reading, predictive processing is a manifestation of what Anderson (2014) calls the “reconstructive” understanding of cognition that we saw in Sect. 2.3, in which the function of the perceptual system is to transition from an impoverished sensory input to an objective reconstruction of the distal environment.

Nevertheless, this interpretation is subtly but importantly misleading. As many authors have pointed out, predictive processing does not make representation *itself* an *end* of brain function (Clark 2016, 168; Hohwy 2013, 55; Seth 2015). Instead, phenomena such as perception, learning and action are better and more perplexingly viewed as *emergent* from a deeper imperative to minimize prediction error.

To understand this, one must situate predictive processing within the context of the “free-energy principle,” an ambitious framework in theoretical biology and neuroscience in which prediction error minimization is viewed as a special case of a more fundamental imperative in biological systems to self-organize under conditions tending towards increasing disorder (Friston 2009, 2010; Friston and Stephan 2007). Crucially, this theoretical context situates predictive processing within the control-theoretic framework for understanding brain function advanced by advocates of “action-oriented” cognition that we saw in Sect. 2.3. As before, any *evaluation* of the free-energy principle lies far beyond the scope of the current paper. Instead I provide a heuristic overview of those of its features that are necessary for general understanding and that bear on my interests here.

The free-energy principle begins with the familiar observation that biological systems are distinctive in acting upon their environments to maintain their structural integrity and the homeostasis of their essential variables, thereby appearing to violate the increasing tendency towards entropy mandated by the second law of thermodynamics (Friston 2009; Schrödinger 1945). In other words, biological systems restrict themselves to a narrowly circumscribed subset of possible biophysical states and thus maintain homeostasis over long (but finite) timescales (Friston 2009). There are an enormous number of states a rabbit *could* be in, most of which would be inconsistent with its survival. Somehow it remains within a subset of such states, reflecting the nature of its phenotype and what’s required for that phenotype to remain viable.

Importantly, these states can be described in terms of the environment’s impact upon the biological system—that is, activity at and transitions between the states at its sensory interface with the world (Friston 2010). Thus if we consider an organism’s phenotype an implicit model of the set of states it must remain within to survive, homeostasis can be glossed as the minimization of *surprisal*, where “surprisal” is an information-theoretic term that measures the improbability of an outcome relative to a model. Crucially, this makes surprisal *organism*-*relative*: what has high surprisal for one organism may have low surprisal for another (Hohwy 2013, 52). In effect, this means that biological systems are “defined by the particular way they resist disorder,” such that “a specific type of living agent simply *is* a set of states that maintain themselves within certain bounds—the bounds that describe the conditions necessary for their own survival” (Clark 2017, 3).

Biological systems cannot directly evaluate the surprisal of a given state, however, for they can’t average over an infinite number of copies of themselves in all possible states to evaluate the surprisal of a given sensory state (Hohwy 2015, 3). This fact then motivates Friston’s (2009, 2010) bold proposal: a tractable optimization task that the brains of organisms *can* perform that *approximates* the minimization of surprisal is the minimization of *variational free energy*—an information-theoretic quantity that, under some simplifying assumptions, translates to *long*-*term prediction error*. Thus “prediction error minimization is, essentially, a tool for self-organisation” (Gladziejewski 2015, 563).

As the authors of a recent textbook put it, “the core task of all brains… is to regulate the organism’s internal milieu” (Sterling and Laughlin 2015, xvi). Predictive processing is advanced as a “process theory” (Friston et al. 2017) intended to explain how they achieve this.

For our purposes, what is crucial about the free-energy principle is its emphasis on the extent to which *internal representation* is understood within the context of predictive processing *as a means* to a *non*-*representational, pragmatic end*: namely, the end of maintaining the homeostatic integrity of the organism under conditions tending towards increasing disorder. This effectively situates predictive processing in the context of the “good regulator theorem” advanced in the cybernetics tradition (Seth 2015), and implies that the brain recovers the distal environment through its generative model only insofar as it bears on its regulatory function—an important point I return to below.^17^ As Seth (2015, 3) puts it, “perception emerges as a *consequence* of a more fundamental imperative towards homeostasis and control, and not as a process designed to furnish a detailed inner “world model” suitable for cognition and action planning” (Seth 2015, 3, my emphasis).

### Modelling the Umwelt

An immediate upshot of this pragmatic perspective on brain function is that the *contents* of generative models within predictive processing are profoundly *organism*-*relative*, structured by the contingent practical interests and idiosyncratic properties of the organisms of which they are a part (Clark 2015, 2016; Madary 2015; Barrett 2017a). As Gladziejewski (2015) puts it, “the way the whole prediction error minimization machinery works is not neutral from the point of view of the “interests” of an organism as a self-organising entity.”

To see this, recall again the core tenets of the reconstructive perspective on cognition introduced in Sect. 2. On this view, the brain can be decomposed into functionally differentiated perceptual sub-systems that implement algorithms for computing the value of functions, where the output—the value—of such functions is understood as a veridical representation of the distal environment to be passed on to “higher” cognitive areas (Crane 2003, ch. 3). Marr's (2010) seminal work on vision provides the exemplar: the purpose of the visual system is to provide an accurate and objective three-dimensional representation of the distal environment—to identify “what is where” (Marr 2010)—from the representation of light intensities on the retina. In addition, the more “discursive” regions of mental representation in classical cognitive science are typically understood to be reliant on a determinate reference mapping from brain-bits to reality-bits, specifiable in the perspective-independent vocabulary of natural science (Fodor 1987; Fodor and Pylyshyn 2015).

The upshot of this “classical representation-centred paradigm” is clear: the brain’s function is at least in part the construction of veridical re-presentations of the world, the contents of which are explained in terms of a mapping between internal and independently identifiable external states. The “subject of cognition” is thus viewed as “a detached observer with a “bird’s eye” view of the world” (Engel et al. 2015, 3).

Predictive processing positions itself in stark opposition to this view. The brain represents the causal structure of the ambient environment only insofar as it bears on its practical function of homeostatic control, extracting “the patterns that matter for the interactions that matter” (Clark 2016, 292) and discarding the rest. As Barrett (2017a, 3) puts it, “a brain did not evolve for rationality, happiness or accurate perception. All brains accomplish the same core task: to efficiently ensure resources for physiological systems with an animal’s body (i.e. its internal milieu) so that an animal can grow, survive and reproduce.” The upshot is straightforward, profound, and diametrically opposed to reconstructive views: “modelling the world “accurately” in some detached, disembodied manner would be metabolically reckless. Instead, the brain models the world from the perspective of its body’s physiological needs” (Barrett 2017a, 6).

In the vocabulary introduced in Sect. 2, the brain thus recovers the organism’s “Umwelt,” or what Barrett (2017a, b) nicely terms its “*affective niche*”: the environment as it *matters* to the organism and its physical integrity. “Anything outside your affective niche,” Barrett (2017b, 73) notes, “is just *noise*: your brain issues no predictions about it, and you do not notice it.”

In a wonderful essay on the “frame problem” in classical artificial intelligence, Haugeland (1987, 92) expresses a worry with cognitive-scientific theories that rest on internal models of the sort that predictive processing postulates:

One thing that’s frightening about “mental scale models” is that there’s no obvious end to them: Why not just recreate the entire universe, monad-like, inside each individual brain? Well, because it’s manifestly absurd, that’s why. But what could motivate, or even delineate a more modest scheme?

Predictive processing motivates a more modest scheme: generative models recapitulate the causal-probabilistic structure of the organism’s affective niche as carved out by the brain’s regulatory function. As Clark (2016, 196) puts it, it is “the *agent*-*salient* structure of the distal realm [that] becomes reflected in both the large-scale shape and the spontaneous activity patterns of the neural architecture” (my emphasis). The rest is discarded as noise.

### Summary

Predictive processing presents a radical and exciting conception of cognitive activity: brains are prediction machines that self-organize around the imperative to minimize the mismatch between predicted and received sensory inputs, an imperative that mandates both reactive and active inference. These inferential processes are made possible through the construction of a richly structured hierarchical generative model of functionally salient environmental causes, the contents of which are coloured at every level by the practical interests of the organism and function as instruments in the service of homeostatic control.

## PP and the Representation Wars

My thesis is this: by nesting a compelling structural resemblance-based account of internal representation within a fundamentally pragmatic brain, predictive processing has the resources to either avoid or accommodate the chief anti-representationalist concerns outlined in Sect. 2. I now consider each of these challenges in turn.

### Representational Function

Recall the first anti-representationalist challenge introduced in Sect. 2: do the structures characterised as representations in the foregoing presentation—as “inferences,” “predictions,” and “generative models,” for example—genuinely *warrant* this representational interpretation? That is, do they perform recognisably representational jobs within the cognitive architecture described by predictive processing?

Anderson and Chemero (2013) have recently expressed scepticism on just this score. They argue that representational interpretations of predictive processing conflate “different senses of “prediction” that ought to be kept separate.” One sense of “prediction”—what they call “prediction1”—“is closely allied with the notion of correlation, as when we commonly say that the value of one variable “predicts” another,” and is “essentially model-free” (Anderson and Chemero 2013, 203). Another sense (“prediction2”), by contrast, “is allied instead with abductive inference and hypothesis testing,” and is “theory laden and model-rich.” At most, they argue, the evidence for predictive processing is evidence for the ubiquity of prediction1 in cortical activity. Conceptualising such activity in terms of prediction2 is a “theoretical choice not necessitated by the evidence” (Anderson and Chemero 2013, 204). Given that one *can* describe the functional asymmetry between bottom-up and top-down signals at the core of predictive processing in a non-representational vocabulary,^18^ Anderson and Chemero raise a reasonable challenge: why *bother* with the representational interpretation of such cortical processes advanced above?

This challenge is easily answered, however. As Gladziejewski (2015) has recently argued, the generative models posited by predictive processing perform robustly representational functions within the overall cognitive architecture it posits. Indeed, predictive processing “might be as representational as cognitive-scientific theories get” (Gladziejewski 2015, 561).

I won’t recapitulate every detail of Gladziejewski’s nuanced treatment here, with which I am in complete agreement. For our purposes, the core idea is relatively straightforward: generative models within predictive brains function as “*action*-*guiding, detachable, structural models* that afford *representational error detection*” (Gladziejewski 2015, 559). Each of these characteristics should be familiar from the foregoing presentation, so I will move through them relatively quickly.

First, generative models are *structural models* in exactly the sense introduced in Sect. 3.3: they are physically realised cortical networks that recapitulate the causal-probabilistic *structure* of the (functionally significant) environment.

Second, this structural resemblance is actively *exploited* by the brain in its proper functioning, guiding the organism’s environmental interventions. To see this, recall from Sect. 3 *why* brains minimize prediction error: namely, to maintain the organism within its expected states. As Gladziejewski (2015) notes, the central thesis of predictive processing is that the brain’s ability to achieve this feat is *dependent* on the resemblance between the causal-probabilistic structure of the generative model and the ambient environment. That is, effective active inference is only possible given a sufficiently accurate model of the causal-probabilistic dependence relationships among significant environmental variables (cf. Hohwy 2013, 91). As Gladziejewski and Milkowski (2017) note in a recent paper, this makes the structural resemblance between the generative model and the environment *causally relevant* to the brain’s proper functioning. Such models are thus “action-guiding” in that the organism’s ability to intervene on its environment to maintain its viability is functionally *dependent* on the degree to which its cortical networks accurately recapitulate the causal-probabilistic structure of the action-relevant environment.

Third, an implication of this is that such models are “detachable.*”* Specifically, it is the generative model *itself* that functions as the locus of behavioural control—of the organism’s active-inference induced environmental interventions—and *not* some direct coupling with the environment. As Gladziejewski (2015) puts it, “active inferences are dictated by *endogenously*-*generated* hypotheses about causes in the external world.” In this way such generative models genuinely function as a *proxy* or *stand*-*in* for the surrounding environment in much the same way that one might exploit a *map* as the locus of *navigational decisions* in navigating an unfamiliar terrain. Further, given the fundamentally *predictive* character of generative models, this detachment is such that active inferences are guided as much by model-based *expectations* (predictive simulations) of how things *will be* as by estimates of how they *are*.

Finally, such generative models afford *representational error detection*. Specifically, they enable the brain to determine to what extent *its* internal stand-in for the environment genuinely mirrors its functionally relevant causal structure. This follows from a simple fact: because the brain’s proper functioning is dependent on its ability to minimize prediction error, and this ability is in turn dependent on to what extent its internal model recapitulates the causal-probabilistic structure of the world, the brain can harness failures of prediction error to detect errors in the accuracy of its internal model. Indeed, it is this ability of predictive brains to harness their own sensory inputs as *feedback* to the installation and deployment of their generative model that is one of the most attractive features of predictive processing (Hohwy 2013, 49).

As this analysis showcases, the characterisation of generative models as *models* within predictive processing is neither idle nor vacuous. Such structures function in a way that is robustly representational in character, enabling brains to effectively coordinate the organism’s behaviour with the surrounding environment by constructing an internal surrogate or simulation of that environment with which to predict its sensory effects and support adaptive interventions. It is thus not *just* that cortical networks recapitulate the causal-probabilistic structure of the environment that renders them generative models. It is the fact this structural resemblance is causally relevant to the brain’s homeostatic functioning and exploited in a way that is recognisably representational in character. Talk of “models” and “prediction” is therefore fully justified.

With this analysis in hand, consider again Anderson and Chemero’s preference for focusing exclusively on anticipatory dynamics within cortical networks in place of the representational interpretation advanced here. It should now be clear that this suggestion neglects the two most important questions in the vicinity. First, what is the *function* of such anticipatory dynamics? Second, *how* are they *achieved*? It is in answering *these* questions that the representationalist interpretation of predictive processing is required: effectively anticipating the incoming signal is necessary for the organism’s ability to intervene upon the environment to maintain homeostasis, and it is made possible by the exploitation of an internal *model* of the signal *source*. Without this representationalist interpretation, the brain’s ability to so successfully “predict1” its incoming sensory inputs is both *unmotivated* and *unexplained*. It is not enough to show *that* brains are “prediction machines”: predictive processing explains *how* and *why* they become this way—namely, by installing and deploying a model with which to guide the organism’s viability-preserving interventions in the world.

### Content Determination

Recall now the second challenge introduced in Sect. 2: representational content cannot find a place in the natural world. After consciousness, this “problem of intentionality” constitutes the most significant challenge to a thoroughly naturalistic understanding of the mind, and it has given rise to a truly staggering amount of philosophical work. Of course, I cannot demonstrate that predictive processing solves this perennial problem here. Instead, I offer some preliminary reasons to think that it genuinely transforms the *nature* of the problem in a significant way. Specifically, I argue that it situates the problem firmly in the domain of *cognitive science*, not *metaphysics*.

To see this, it is helpful to begin with a remark by Clark (2015, 2) in a recent paper discussing the implications of predictive processing for the problem of content:

To naturalize intentionality… “all” we need do is display the mechanisms by which such ongoing viability-preserving engagements are enabled, and make intelligible that such mechanisms can deliver the rich and varied grip upon the world that we humans enjoy. This, of course, is exactly what PP [predictive processing] sets out to achieve.

This passage should be puzzling for two reasons. First, Clark seems to suggest that naturalizing intentionality is a matter of identifying the *neural mechanisms* implicated in hierarchical prediction error minimization, which he takes to be part and parcel of the first-order research programme of predictive processing itself. This stands in stark contrast to the division of labour philosophers are accustomed to, in which cognitive scientists posit a computational architecture and philosophers explain what determines the contents of its representations (Fodor 1987; Von Eckardt 2012). Second, Clark seems to ignore all those characteristics of intentionality that have made the problem of content so difficult, reducing it instead to our ability to gain a “rich and varied grip upon the world.” What about determinacy, shared contents, and the possibility of *mis*representation, for example (Fodor 1987; Hutto and Satne 2015)? It is common knowledge in the philosophy of mind that a mere account of internal mechanisms has little to say about such recalcitrant phenomena.

Nevertheless, I think that Clark is on to something, and it follows once more from predictive processing’s structuralist approach to internal representation.

First, recall from Sect. 2.2 that almost all work on “naturalizing content” has been concerned with linguaformal semantic properties, where the challenge has been to establish the referential properties of in-the-head symbols from which the propositional contents (truth-conditions) of intentional states are recursively constructed. At the heart of this project is a rigid distinction between the formal or “syntactic” properties of such symbol structures and their semantic properties, in which—as with all forms of digital computation—it is assumed that computational procedures are sensitive only to the former, not the latter. Those who argue that *cognition* is a matter of syntax-sensitive operations on symbol structures thus need a story about how such structures acquire their contents—hence the project of “naturalistic psychosemantics” (Fodor 1987). As many have noted, however, a worry with this project is that its *starts* from the view that the representational status of such structures is epiphenomenal. Worse, this worry is exacerbated by the fact that most attempts to provide a semantics for such symbol structures appeal to *extrinsic* properties such as causal or informational relations that are irrelevant to the intrinsic properties by which they perform their functional roles (Bechtel 2009; O’Brien and Opie). For many, this engenders the suspicion that such forms of in-the-head digital computation are not truly representational at all (Stich 1983; Searle 1980), or that their semantic interpretation is at best part of the “informal presentation of the theory” (Chomsky 1995, 55)—what Dennett (1987, 350) once called a “heuristic overlay” (cf. also Bechtel 2009; Egan 2013).

Structuralist accounts of internal representation of the sort implied by predictive processing fundamentally transform this situation in at least two important ways. First, the semantic properties of such models are grounded in their *intrinsic structure*—in the case of predictive processing, in the intrinsic patterns of cortical activity that realise its causal-probabilistic structure (Cummins 2010). Thus the properties implicated in cognitive *processing*—the intrinsic structure of the representational vehicles—are the *same* properties in virtue of which they represent (through resemblance) their target (O’Brien and Opie 2010). Second, as noted in the previous section, this structural resemblance between the two systems is *causally relevant* to the cognitive system’s functioning: the proper functioning of predictive brains is causally dependent on the structural resemblance between their generative model and the environment (Gladziejewski and Milkowski 2017). These two features are bound up with one another, of course: it is only *because *the intrinsic structure of a predictive brain's internal model is simultaneously responsible both for its ability to represent *and * for the capacities it exhibits that the former can be causally relevant to the latter.

The implication of these facts is straightforward and genuinely transformative, however: issues concerning content determination become directly relevant to the question of how such structures perform their systemic role. As O’Brien and Opie (2010) note, representational systems that exploit a structural similarity between their internal states and their target are not merely “syntactic engines” that acquire a semantics through *interpretation* or through hypothesised causal relations to environmental states; they are full-blown “*semantic* engines” in which “computational processes … are driven by the very properties that determine the contents of [their internal] vehicles” (O’Brien and Opie 2010, 8).

The immediate implication of this fact is to situate questions concerning content determination firmly in the realm of cognitive neuroscience, just as Clark suggests. The question becomes *how* the brain’s structural and dynamical properties can recapitulate the nested causal structure of the environment in the exploitable manner suggested above—a question upon which there has already been extensive research (Friston 2002, 2008). The problem of integrating representational properties into a scientific metaphysics thus becomes first and foremost a problem in *science*, not *metaphysics*. Of course, the suggestion is not that philosophers have no role to play in this project—a self-defeating suggestion in the current context, and one undermined by the recent explosion of extremely valuable work in just this area drawn upon here.^19^ Rather, the claim is that this work is now firmly entangled with the explanatory concerns of first-order science in a manner largely *absent* from the programme of naturalistic psychosemantics as it has been practiced in recent decades.^20^

But what about those desiderata that have proven so difficult to accommodate in this project: determinacy, shared contents, the possibility of misrepresentation, and so on? How would a mere account of *neural mechanisms* speak to *those* phenomena?

This gets things backwards, however. Cognitive science—indeed, science in general—is under no obligation to accommodate folk psychological or semantic intuitions (Churchland 2012; Cummins 2010). *Contra* Hutto (2017), the mere fact (if it is a fact) that we currently have no story about how to reduce semantic properties as viewed through the lens of folk psychological intuition—namely, as fine-grained determinate truth-conditions—to purely physical properties is not *itself* an objection to representationalist treatments of predictive processing. The question is whether such properties are necessary for generative models to perform their functional role. And—as a number of philosophers have noted (Churchland 2012; Cummins 2010; O’Brien and Opie 2015)—these properties in fact sit uneasily with structural representations of the sort harnessed by predictive brains. Representational media such as maps and models, for example, typically lack the fine-grained, determinate contents we pre-theoretically attribute to folk psychological states and associate with linguistic expressions, and these characteristics are likely to be carried over to representation in natural systems.^21^ Further, the prospects of identical or shared contents looks hopeless in the context of predictive processing: the internal models of similar animals with similar learning histories will no doubt overlap and resemble each other to substantial degrees, but their contents will still likely be endlessly idiosyncratic (Clark 2015).

What about the notorious problem of *misrepresentation* or *error*? Again, I cannot hope to tackle this enormous issue here, except to note one cause for optimism: by focusing on the subservience of generative models to pragmatic success, predictive processing moves us away from a picture of internal representations as *judgements* to one in which they function as representational *tools*—that is, physically instantiated surrogates for the action-salient causal structure of the environment that facilitate viability-preserving environmental interventions. As many have noted, structural representations force us to shun the idea of representational evaluation as a *binary* phenomenon in favour of a much looser and more graded notion of accuracy or “aptness,” where—crucially—the vehicle’s *representational value* is relativized to the sort of practical application for which it exists to provide guidance (Horst 2016, 86).^22^ It is a familiar theme in the philosophy of science that *models* are not *true* or *false*; they are invariably highly idealised, selective and often purposefully distortive *stand*-*ins* for a domain that enable us to coordinate our practical engagements with it (Giere 2004). Representational *error* must therefore be evaluated against such practical ends. As Clark (2015, 4) puts it, “the *test* of a good [generative] model is how well it enables the organism to engage the world in a rolling cycle of actions that maintain it within a window of viability” (my emphasis).

If this is right, it suggests that many of the problems associated with classical attempts to naturalize intentionality may not arise in the context of predictive processing. Clark’s suggestion is perhaps a little over-stated, but it touches on something important. The core thesis of predictive processing is that brains install and deploy a generative model of environmental causes in the service of homeostasis. If we can explain *how* cortical networks come to embody these pragmatic structural models, and how such models can be exploited in cognitive functioning, we will have “naturalized” intentionality in the only way that could be important to the representational status of the framework.

Before turning to the final challenge outlined in Sect. 2, it is worth introducing an objection that might naturally arise in response to the foregoing treatment. The objection is this: even if one accepts that predictive processing can avoid the first two anti-representationalist challenges in the manner I have suggested, the principal explanation of this is not anything specific to *predictive processing*. Rather, it is the fact that predictive processing posits *structural representations*. Such structural representations, however, are common to a much broader class of approaches in cognitive science, including both classical computational and connectionist accounts of information-processing. Thus it is not predictive processing *as such* that puts an end to the representation wars, but the broader class of structural model-based approaches of which it is merely one manifestation.^23^

This objection clearly gets *something* right. A structural approach to internal representation has become increasingly popular in recent years—and for good reason.^24^ Part of the argument I have advanced here is that predictive processing can *capitalize* on the theoretical advantages it enjoys with this broader class of models.

Nevertheless, predictive processing also contributes something genuinely novel. In addition to its implication that model-based representation is the *fundamental* kind of representation employed by the brain, it also *situates* this compelling structural resemblance-based account of internal representation within an overarching account of neural function that can effectively answer the *third* anti-representationalist challenge introduced in Sect. 2. It thus comes with a fuller package of answers to the concerns raised by those sceptical of internal representations in cognitive science. It is to this final challenge, then, that I turn next.

### Cognitive Function

Superficially, at least, the third anti-representationalist challenge introduced in Sect. 2 is the most straightforward to address given the presentation of predictive processing in this paper. This challenge, recall, contends that the concept of representation implies an implausibly “reconstructive” account of perception that fails to capture the “action-oriented” character of cognition and thus the many profound ways in which contingent properties of the *organism* are implicated in the contents of its experience.

First, predictive processing fully embraces the control-theoretic perspective on brain function we saw associated with the most perspicuous advocates of this “action-oriented” view in Sect. 2. Predictive brains are fundamentally *pragmatic brains*, designed to maintain the organism’s viability under conditions tending towards disorder. As we saw in Sect. 3.4, any *representation* that occurs in such systems is subservient to this practical end.

In addition, numerous authors have noted that predictive processing provides a literal vindication of the functional primacy many in the EEEE tradition ascribe to *action* in cognition (Bruineberg et al. 2016; Clark 2016). To see this, note that *reactive* or *perceptual inference*—that is, the process by which brains update top-down predictions to bring them into alignment with the incoming signal—is *in itself* impotent when it comes to minimizing “surprisal,” the ultimate function of prediction error minimization. As Hohwy (2013, 85) nicely puts it, “perceptual inference can make you perceive that you are hurtling towards the bottom of the sea… but cannot do anything to change that disturbing sensory input.” It is only through *active inference* that organisms can intervene upon their environments to actively minimize surprising exchanges with them. Thus “perception plays a secondary role in optimising action” (Friston and Stephan 2007, 418), just as many advocates of embodied cognition have long argued (Engel et al. 2015; Glenberg et al. 2013).

Perhaps most importantly, however, predictive processing accommodates the hostility towards “reconstructive” accounts of perception expressed by those in the EEEE tradition. As noted in Sect. 3.5, the world modelled by predictive brains is the organism’s *affective niche*, the causal-probabilistic structure of the environment *as it bears upon the brain’s regulatory function* and thus the organism’s physiological integrity. This concept of an “affective niche” can accommodate metaphors like “enacting a world” and “world-making” in the enactivist tradition within a thoroughly representationalist outlook on cognition. Indeed, as Barrett (2017b, 83) puts it (characterising homeostasis as the maintenance of one’s “body budget”): “from the perspective of your brain, anything in your affective niche could potentially influence your body budget, and nothing else in the universe matters. That means, in effect, that *you construct the environment in which you live*.”

Nevertheless, at this point a potential objection raises its head. If the contents of these generative models are as profoundly *organism*-*relative* as I have suggested, what sense can be made of the structural *resemblance* that has been at the core of the view advanced here? That is, is there any prospect of independently identifying “what stands on the other side” of this alleged resemblance relation?^25^ If *not*, one might object that talk of *re*-*presentation* is not warranted: perhaps this thoroughly pragmatic perspective on brain function should force us to ditch such reconstructive talk in favour of a “performative” or “enactive” understanding of the mind. Bruineberg et al. (2016, 15) suggest as much in their anti-representationalist treatment of predictive processing: “if my brain is a scientist,” they argue, “it is a crooked and fraudulent scientist.” Their worry is that cortical networks do not really *recapitulate* the objective causal-probabilistic structure of the external environment: they are “vehicles of world-making”, not “world-mirroring devices” (Engel et al. 2015, 5).

There are two reasons this objection is misguided.

First, the fact that a model is not “neutrally specified” or “observer-independent” (Anderson 2014) does not imply it is not a model. Advocates of EEEE cognition often write as if the only kind of viable internal representations are what Clark (2001) calls “objectivist representations,” namely perspective-independent representations of the action-neutral environment of the sort familiar from models of perception in classical cognitive science. This cannot be right, however. Most if not all models in *science* are heavily idealised, partially distortive and interest-relative (Giere 2004; Horst 2016). The question is whether the relevant vehicle or vehicles are being exploited as a structural surrogate for another domain, and we have seen excellent reason to suppose they *are* in the case of predictive processing: predictive brains exploit cortical activity as a stand-in for the ambient environment with which to anticipate its sensory effects and support viability-preserving interventions.

Second, the organism-relativity defended here does not imply that the elements of generative models are *imagined*. It is vastly implausible that brains could generate time-pressured and adaptively valuable behaviour in hostile environments without at least partially recovering the objective structure of such environments. As Gibson (1979) himself stressed, “affordances” are not *subjective*. The point is rather that the objective structure predictive brains *do* recover is interest-relative and specified relative to the organism’s practical abilities for intervention. In Anderson’s (2014, 2016) “performative” theory of brain function, he writes that “because perception is both active and in the service of action, much of the information to which organism are attuned is not objective information of the sort one might need for model-building, but rather *relational* information that is more immediately useful for guiding action in the world” (Anderson 2016, 7). The contrast here is simply confused, however: *relational information*—for example, the network of complex dependence relationships between essential organismic variables, environmental states and opportunities for intervention—is perfectly *objective* and *exactly* the kind of information *structural* models are suited to represent.

The upshot of these considerations is that predictive processing can accommodate what is *important* in the third anti-representationalist challenge introduced in Sect. 2 while nevertheless preserving its robustly representational status. Predictive brains are not passive spectators: they are vehicles of pragmatic success, facilitating self-organization through the construction and exploitation of structural stand-ins for the organism’s affective niche.

### Summary

If the foregoing arguments are along the right lines, Clark’s hopeful prophecy of a satisfying peace in the representation wars of recent decades is warranted: by nesting a compelling structural resemblance-based account of internal representation within a fundamentally pragmatic brain, predictive processing has the resources to either embrace or avoid the most serious concerns raised by anti-representationalists without foregoing a fundamentally model-based approach to perception, cognition and action. In one of the most sophisticated broadly anti-representationalist tracts in recent time, for example, Anderson (2014, 162) writes that his chief objection to the postulation of internal representations “is that it comes freighted with the baggage of reconstructive perception and the symbol systems hypothesis.” Predictive processing, however, comes with neither. In place of the formally individuated symbol structures and syntax-sensitive operations characteristic of classical cognitive science, it advances a cortically realised network of causal-probabilistic maps. And in place of an image of minds as “mirrors of nature,” it advances an action-oriented recapitulation of an organism’s idiosyncratic Umwelt—a representation of the environment as it *matters* to the organism in facilitating the ultimate kind of pragmatic coping.

## Conclusion

Much more work needs to be done, of course. Many of the foregoing claims have been schematic at best and would require substantial elaboration in a longer and more extensive treatment. How does the *probabilistic* and *Bayesian* component fall out of the structural resemblance interpretation of generative models, for example?^26^ How can a model-based architecture as advanced by predictive processing accommodate the symbolic and propositional kinds of representation with which we are familiar?^27^ What are the implications of the foregoing account for our folk-psychological commitments to *beliefs* and *desires* and the semantic characteristics we pre-theoretically attribute to such states? Further, I have said nothing about many of the more *philosophical* dimensions that have characterised the debate between representationalists and their opponents: the mind/world relation, epistemic internalism, and Kantian projectivism, for example.^28^ Perhaps most importantly, I have ignored all empirical questions concerning the explanatory credentials of predictive processing, and it has recently received some impressive and withering critiques (Colombo and Wright 2016; Klein 2016).

Nevertheless, I hope the present paper has advanced the fascinating contemporary debate about the nature of internal representation within predictive processing, and offered some additional support for the exciting prospect that it might herald a unifying framework for the integration of important insights from intellectual traditions commonly understood as rivals.

## Footnotes

1 Shapiro (2011) provides an excellent overview.

2 There is an enormous literature tackling this question (cf. Haugeland 1991; Ramsey 2007; Von Eckardt 2012).

3 The explanatory irrelevance of meaning is also central to Quine’s (1960) anti-representationalism (c.f. Hylton 2007). See also Bechtel (2009) for this view of classical computationalism.

4 This seems to be the view of Peirce (1931–58), Ryle (1949), and Wittgenstein and Anscombe (1953).

5 See Ramsey (2007, ch. 4) for an up-to-date version of this challenge.

6 Ramsey (2007) argues that only classical computationalism passes the job description challenge, partly in virtue of positing structural or “S-representations”. If my argument here is correct, predictive processing does as well—and (partly) for the same reason (see Sect. 4.1 below).

7 See Hutto and Satne (2015) for an up-to-date review.

8 Quoted in Clark (2016, 290).

9 See especially Clark (2013, 2016), Friston (2009, 2010), Hohwy (2013), and Seth (2015).

10 See Gibson (1979) and Anderson (2017) for challenges to this starting assumption.

11 Clark (e.g. 2016, 309, fn. 19) notes the non-linguaformal character of predictive processing representations, but not (explicitly) their *analogue* character (see below).

12 See also Gopnik et al (2004).

13 The structural resemblance is “second-order” in that it doesn’t require the *kinds* of relations to be the same, only that the *pattern* of relations among elements in the one domain replicates the pattern of relationships among elements in the other.

14 See also Grush (2004).

15 For the former, see Gladziejewski (2015) and Wiese (2016). For the latter, see Churchland (2012). A fascinating question here is how to make sense of representational activity underlying *active inference*, especially the counterfactual predictions involved (cf. Gladziejewski 2015, 575, fn. 14).

16 Of course, this raises the fascinating question of how such a model architecture might be transformed, augmented and restructured by exposure to the vehicles of natural language in humans. See Clark (2016, ch. 10) for exploration of this question, and Churchland (2012, ch. 5) for discussion of language in the more general context of analogue cognition.

17 The theorem states that "every good regulator of a system must be a model of that system," thus essentially tying *homeostasis *to *representation* (Conant and Ashby 1970).

18 See Rao and Ballard (1999). Indeed, it is this fact that makes the predictive processing architecture a genuine “process theory” with a potential mechanistic implementation.

19 I thank an anonymous reviewer for pressing this point.

20 See Cummins (2010) for this criticism.

21 I thank an anonymous reviewer for this way of framing things.

22 Gladziejewski (2015, fn. 6) make a similar point.

23 I thank an anonymous reviewer for forcefully articulating this objection.

24 See Gladziejewski and Milkowski (2017) for an excellent overview and analysis.

25 I thank an anonymous reviewer for pressing this point.

26 See Gladziejewski (2015) and Wiese (2016) for some tentative work in this area.

27 Clark (2016, ch. 9) and Ramstead et al. (2016) take up this question.

28 These themes feature heavily in Chemero’s and Anderson’s (2013) critique of predictive processing, for example.
